# CXCL16 and CXCR6 Are Coexpressed in Human Lung Cancer *In Vivo* and Mediate the Invasion of Lung Cancer Cell Lines *In Vitro*

**DOI:** 10.1371/journal.pone.0099056

**Published:** 2014-06-04

**Authors:** Weidong Hu, Yue Liu, Wenhui Zhou, Lianlian Si, Liang Ren

**Affiliations:** 1 Department of Thoracic Oncology, Zhongnan Hospital of Wuhan University, Wuhan, People’s Republic of China; 2 Hubei Cancer Clinical Study Center & Hubei Key Laboratory of Tumor Biological Behaviors, Wuhan, People’s Republic of China; 3 Reproductive Medicine Center, Beijing Chao-Yang Hospital of Capital Medical University, Beijing, People’s Republic of China; 4 Reproductive Medicine Center, Zhongnan Hospital of Wuhan University, Wuhan, People’s Republic of China; Cincinnati Children's Hospital Medical Center, United States of America

## Abstract

Despite advances in early diagnosis and multimodality therapy for cancers, most of lung cancer patients have been locally advanced or metastatic at the time of diagnosis, suggesting the highly progressive characteristic of lung cancer cells. The mechanisms underling invasiveness and metastasis of lung cancer are yet to be elucidated. In the present study, immunohistochemistry was performed to detect the expression of CXCL16-CXCR6 in human lung cancer tissues. It was demonstrated that similar to CXCL12 and CXCR4, CXCL16 and CXCR6 were also coexpressed in human primary lung cancer tissues. After confirming the functional existence of CXCL16 and CXCR6 protein in A549, 95D and H292 cells by ELSA and flow cytometry analysis, we further explored the significance of CXCL16-CXCR6 axis in the biological functions of lung cancer cell lines *in vitro*. It was found that CXCL16 had no effects on the PCNA (proliferating cell nuclear antigen) expression of A549, 95D and H292 cells. However, both exogenous CXCL16 and CM (conditioned medium from A549, 95D or H292) significantly improved the *in vitro* viability and invasion of three lung cancer cell lines. The neutralizing antibody to CXCL16 or down-regulation of CXCR6 was able to inhibit the increased viability and invasiveness of A549, 95D and H292 cells stimulated by CXCL16 or CM. Our results imply that CXCL16-CXCR6 axis is involved in the regulation of viability and invasion rather than PCNA expression of lung caner cells, which opens the door for better understanding the mechanisms of lung tumor progression and metastasis.

## Introduction

Lung cancer is a common malignant tumor which ranks as the leading cause of malignancy-related death worldwide, and the incidence of lung cancer has been increasing in recent years in some big cities in China [1]. Despite advances in early diagnosis and multimodality therapy for cancers, the five-year overall survival rate for most advanced lung cancer patients is still less than 20%. The mechanisms underling invasiveness and metastasis of lung cancer have attracted a lot of attentions from thoracic oncologists for decades of years. Some hypotheses and molecules have been put forward, but a thorough understanding of the intricate invasive and metastatic processes of carcinoma cells is still an open question.

Since the discovery of the first chemokine in 1987, more than 50 kinds of chemokines and 20 kinds of chemokine receptors have been cloned and identified. Activation of chemokine/receptor signal pathway has been confirmed to mediate a series of physiological and pathological events, especially the recruitment of lymphocyte as well as tumor growth and metastatic spread, which provides the possibility for the elucidation of metastatic process of malignant cells from the immunology perspectives [2], [3], [4], [5], [6].

Among various chemokines and chemokine receptors, CXCL16-CXCR6 is an unique chemokine/chemokine receptor pair. CXCL16, also known as SR-PSOX, belongs to CXC chemokine family and exists both in a transmembrane and soluble form [7], [8], [9]. Interaction between CXCL16 and its’ sole receptor, CXCR6 (also called Bonzo, STRL33 and TYMSTR) is involved in multiple biological activities, including selective trafficking of lymphocyte subsets, cell adhesion, cell survival, muscle regeneration, brain development, chronic inflammation and anti-tumor immunity [9], [10], [11], [12], [13], [14]. In particular, recent studies have verified the over-expression of CXCL16 and/or CXCR6 in several types of human cancers and CXCL16 could stimulate the growth, migration, invasion and activation of AKT signaling pathway of cancer cells via its’ receptor CXCR6 *in vitro* [15], [16], [17], [18]. Our previous study also confirmed the excessive expression of CXCR6 protein in human native prostate cancer cells and the activation of CXCL16-CXCR6 pathway could promote the migration and invasion of PC3 and LNcap cells *in vitro* [19]. These results suggest that CXCL16-CXCR6 may be a novel chemokine ligand/receptor pairs implicated in tumorigenesis and metastasis. Some groups have begun to pay attention to the role of CXCL16-CXCR6 in tumor, however, the relationship between CXCL16-CXCR6 and lung cancer is still elusive and deserves further investigations.

In the present study, the expression of CXCL16 and CXCR6 in human lung cancer tissues and lung cancer cell lines, A549, H292 and 95D, was determined by immunohistochemistry and immunocytochemistry respectively. Then the enzyme-linked immunosorbent assay (ELISA) and flow cytometry were conducted to examine the functional expression of CXCL16 and CXCR6 in three cancer cell lines. To further elucidate the role of CXCL16-CXCR6 axis in lung cancer, we also observed the action of CXCL16 on PCNA (proliferating cell nuclear antigen) expression and invasive ability of A549, H292 and 95D cells. The effects of CXCR6 gene down-regulation by small interfering RNA (siRNA) technology on viability and invasiveness of A549 cells were also determined by MTT and invasion assay. Through exploring the change of biological behaviour of lung cancer cells mediated by CXCL16-CXCR6 axis, we hope to provide insights into better understanding the progression of this aggressive malignant tumor.

## Materials and Methods

### Human Tissue Collection

All procedures involving participants in the study were approved by the Zhongnan Hospital of Wuhan University Human Research Ethics Committee, and the participants had provided their written informed consents.

Human lung cancer (33 cases) and normal (5 cases) tissues were obtained from patients who underwent pulmonary lobe resection or pneumonectomy for cancer or non-cancer lung diseases at Zhongnan Hospital of Wuhan University from 2003 to 2006. The identification of tumor types was performed by two professional pathologists. Of 33 lung cancers, 13 cases were adenocarcinomas (AC), 12 cases were squamous carcinomas (SC), 7 cases were adenosquamous carcinomas (ASC) and 1 case was bronchoalveolar carcinoma (BC). The stages of tumors were estimated according to the Seventh Edition of the new TNM staging system suggested by the international association for the study of lung cancer (IASLC) in 2009. In the 33 samples, 3, 1, 9, 8, 10 and 2 cases were IA, IB, IIA, IIB, IIIA and IIIB, respectively.

### Cell Culture

Lung cancer cell lines A549, H292 and 95D cells were obtained from the Cell Bank of Chinese Academy of Science (Shanghai, China). The cells were cultured in 1640 containing 10% heat-inactivated FBS (Gibco, Grand Island, NY, USA), 10 mM HEPES, 1 mM pyruvic acid sodium, 4.5 g/L glucose, 100 UI/ml penicillin and 100 µg/ml streptomycin. The cells were recovered and passaged for three times, then allowed to grow to confluence for the following experiments.

### Immunohistochemistry

The method for immunohistochemistry was based on our previous procedure [19]. Briefly, the tissues were routinely fixed with formalin and embedded in paraffin. Antigen retrieval was performed and 0.3% H_2_O_2_ in phosphate buffer solution (PBS) was employed to block endogenous peroxidase activity in the sections. After treated with protein blocking serum to block non-specific binding, the sections were incubated overnight at 4°C with mouse anti-human CXCR4 (25 µg/ml), mouse anti-human CXCR6 (25 µg/ml), mouse anti-human CXCL12 (20 µg/ml) and goat anti-human CXCL16 (20 µg/ml) antibodies (from R&D Systems, Abingdon, UK), respectively. A streptavidin/biotin detection reagent kit (Maixin Bio., Fuzhou, China) with 3, 30-diaminobenzidine tetrahydrochloride (DAB) was employed for signal detection and Harris hematoxylin was used as a counter-stain. Mouse or goat isotype IgG (10 µg/ml) (Dingguo Bio Co. Ltd, Shanghai, China) was used as a negative control and human first-trimester villous tissues (5 samples) were used as positive control (13, 19). Scoring was performed blindly using a telepathology system without knowledge of related clinical information (e.g., tumor grade, tumor size or clinical outcome). The immunostaining intensity was observed and scored as no staining (score = 0), light yellow (score = 1), light brown (score = 2) or brown (score = 3). The percentage of positive cells was calculated as <5% (score = 0), 5–25% (score = 1), 26–50% (score = 2), 51–75% (score = 3) or >75% (score = 4). Combining the immunostaining intensity score and the percentage of positive cells, we classified the scoring as follows: <2, negative expression; 2–3, weak expression; 4–5, moderate expression and 6–7 as strong expression [19].

### Immunocytochemistry

After 70–80% confluence of the cells, A549, H292 or 95D cells were digested with 0.25% trypsin (Bio Basic Inco., BBI, Ontario, Canada) containing 0.1% EDTA and seeded at a density of 2×10^5^cells/well in 6-well plates pre-placed with cover slips. After cultured for 48 h, lung cancer cell lines were fixed in 4% formalin for 20 min at room temperature, washed in PBS and permeabilized for 15 min in 0.03% Triton X-100-PBS, then treated with 0.3% H_2_O_2_ to inactivate endogenous peroxidase activity. The following procedure was as described above in the method of immunohistochemistry and human primary cultured trophoblast cells were also used as positive control [13], [19]. The results were analysed by Image-ProPlus 6.0 software and the mean expression density of CXCL16 and CXCR6 in three cell lines was recorded. The experiments were repeated three times.

### Preparation of Cell Cultured Conditioned Medium

The isolated A549, 95D and H292 were seeded in culture bottles (6 ml/bottle) at a density of 1×10^6^/ml, respectively, and cultured continuously for 48 h. The supernatants, namely conditioned medium (CM), were collected and centrifuged at 2000 g, then stored at −80°C. The supernatants from culture medium without cells were also collected as control.

### Enzyme-linked Immunosorbent Assay (ELISA)

Digested A549, H292 and 95D cells were seeded in 24-well plates (500 µl/well) at a density of 5×10^5^/ml, respectively. Supernatants of the cell cultures were collected at 24, 36, 48, 60, 72, 96 and 100 h of culture. Each collected supernatant was stored at −80°C for the ELISA analysis. The amount of CXCL16 in each supernatant was measured by human CXCL16 ELISA kit (Shanghai Westang Bio-Tech Co. Ltd, Shanghai, China), according to the manufacturer’s instructions. The CXCL16 assay kit demonstrated a sensitivity of 40 pg/ml and an intra-assay coefficient of variation less than 12%. The experiments were repeated three times.

### Flow Cytometry Detection for CXCR6 Expression

The membrane expression of CXCR6 protein on A549, H292 and 95D cells was detected by flow cytometry according to our previous method and CXCR4 was also used as control at the same time [20]. Briefly, to protect the membrane localization of the chemokine receptor to the greatest extent possible, the cells, at 70–80% cell confluence, were digested with 0.25% trypsin only for 30–50 s, then blown off gently and washed with PBS [20]. After blocking with 10% FBS, the recovered cells were incubated with mouse anti-human PE (phycoerythrin) -CXCR6 monoclonal antibody (1∶10; R&D Systems, Inc), mouse anti-human PE-CY5-CXCR4 monoclonal antibody (1∶5; eBioscience), mouse PE-IgG2B isotype (R&D Systems, Inc) or mouse PE-CY5-IgG2a isotype (eBioscience), in the recommended usage for 1 h at room temperature in darkness. The cells were then washed twice with 1 ml of PBS by centrifugation at 2000×g for 5 min and analyzed by FACS Calibur flow cytometry (FC500, Beckman-Coulter, USA) and CellQuest software. The cells of 1×10^5^ were counted and the proportion of positive cells was recorded. The experiments were repeated three times.

### PCNA Detection by Flow Cytometry

The isolated A549, H292 and 95D cells were seeded on 6-well plates at a density of 2×10^5 ^cell/well. At 70–80% cell confluence, the lung cancer cell lines were starved for 12 h, then treated with human recombinant CXCL16 (100 ng/mL) (Peprotech,Rocky Hill, NJ, USA) or a combination of CXCL16 with CXCL16 neutralizing antibody (100 ng/mL) (R&D Systems, Inc.) for anther 48 h. Then the cells were digested and collected for the following PCNA (proliferating cell nuclear antigen) examination. Briefly, the cells were treated with 70% ethanol and 0.1% Triton X-100, each for 20 min, then incubated with mouse anti-human PE-PCNA monoclonal antibody (1∶5; eBioscience) or mouse PE-IgG_2a_ isotype (eBioscience) for 1 h at room temperature in the dark [21]. The following examining procedure was as described above in the method of flow cytometry detection for CXCR6 expression. The experiments were repeated three times.

### CXCR6 Silence in A549 Cells

The isolated A549 cells were seeded on 6-well plates at a density of 2×10^5^/ml. At 70–80% confluence, these cells were transfected with phU6/GFP/Neo plasmid containing short hairpin RNA (shRNA) molecules targeted against CXCR6, with the usage of Lipofectamine 2000 (Invitrogen). The sequences for three shRNA oligonucleotides were: (CXCR6-2819-1): 5′-ctGAG GAC AAT TCC AAG ACT T-3′ (sense) and 5′-AAG TCT TGG AAT TGT CCT CAG-3′ (Anti-sense); (CXCR6-2820-2) 5′-ctCAC CAT GAT TGT CTG CTA T-3′ (sense) and 5′-ATA GCA GAC AAT CAT GGT GAG-3′ (Anti-sense); (CXCR6-2821-1) 5′-gcTTG CTC ATC TGG GTG ATA T-3′ (sense) and 5′-ATA TCA CCC AGA TGA GCA AGC-3′ (Anti-sense) (GENECHEM, Shanghai, China). The groups were divided into phU6/GFP/Neo-CXCR6 (CXCR6-shRNA), non-targeting siRNA oligonucleotides negative control (phU6/GFP/Neo, shRNA-control) and blank-control (no any treatment). Stable transfectants were selected by G418 culture at a concentration of 800 µg/ml. The experiments were repeated three times and the silencing efficiency was determined by western blot.

### Cell Viability Assay

The MTT [3-(4,5-dimethylthiazol-2-yl)-2,5-diphenyltetrazolium bromide, Sigma Chemicals] assay was applied to evaluate the effects of CXCL16-CXCR6 on cell viability *in vitro* [21]. The experiments were divided into two steps: firstly, the isolated A549 cells from blank-control, shRNA-control and CXCR6-shRNA (2819-1) groups were seeded in 96-well flat-bottom microplates with a density of 2×10^3^ cells/well and cultured for 24, 48, 72, 96 and 120 h, respectively. Secondly, after starved with 1640 without FCS for 12 h, the cells from blank-control, shRNA-control or CXCR6-shRNA (2819-1) groups were treated with control medium (1640) or CXCL16 at a concentration of 100 ng/mL for 48 h.

MTT reagent (20 µl) was added to each well of 96-well microplates and incubated at 37°C for 4 h. The medium was decanted and 100 µl of DMSO was added to solubilize the reactive crystals. Absorbency was measured at a wavelength of 570 nm on an automatic microplate reader. The samples were run in triplicate and the experiments were repeated three times [21].

### ECM Invasion Assay

The invasive ability of lung cancer cell lines was evaluated objectively by *in vitro* invasion assay based on our previous method [19], [20]. Briefly, the cell culture inserts (8 µm pore size, 6.5 mm diameter; Corning, Corning, NY, USA) coated with 10 µl pure extracellular matrix (ECM) gel (Sigma, St. Louis, MO 63103, American) were placed in a 24-well plate. The experiments were divided into the following two parts: Firstly, the isolated A549, H292 or 95D cells (1×10^5^/200 µl serum-free 1640) were plated in the upper chamber, and treated with CM, CXCL16 (100 ng/ml) and a combination of CM or CXCL16 with CXCL16 neutralization antibody(100 ng/mL). Secondly, the A549 cells, from the blank-control, phU6/GFP/Neo and phU6/GFP/Neo-CXCR6 group, were seeded on the upper chamber at a density of (1×10^5^/200 µl serum-free 1640), then treated with CXCL16 (100 ng/ml) or CM. The lower chambers were filled with 800 µl 1640 medium supplied with 10% FBS. After incubated at 37°C for 24 h, the inserts were removed and washed in PBS. Then the noninvading cells together with ECM gel were removed from the upper surface of the filter by wiping with a cotton bud. The inserts were fixed in 4% formalin for 10 min at room temperature, and stained with hematoxylin. The result was observed under a light microscope (Olympus, Tokyo, Japan) and the cells migrated to the lower surface were counted. To eliminate the individual variability, the results were assessed by two independent researchers and the invasive index was calculated as the proportion of the migrated cells of the experiment group to that of its own control. Each experiment was carried out in triplicate, and repeated three times.

### Statistics

The experiments *in vitro* were shown in the mean ± SE. The data of PCNA expression, *in vitro* viability and invasion assay were assessed with the post hoc Dunnett’s *t* test and Dunnett T3 test, when appropriate. The differences were accepted as significant at *P*<0.05.

## Results

### Protein Expression of CXCL16-CXCR6 in Human Lung Cancer *in vivo*

**Figure 1:**
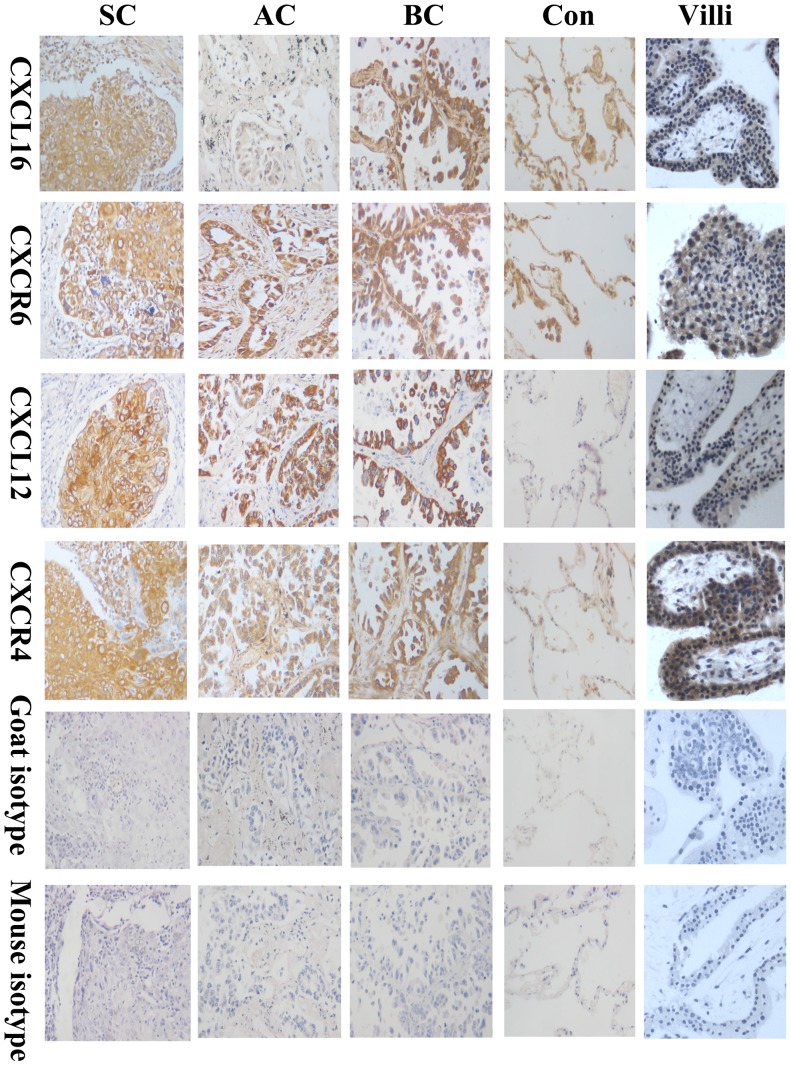
CXCL16-CXCR6 and CXCL12-CXCR4 were co-expressed in human lung cancer. Immunohistochemistry was performed to determine the protein expression of CXCL16-CXCR6 and CXCL12-CXCR4 in human primary lung cancer tissues. The antibodies used in this experiments were mouse anti-human CXCR4 (25 µg/ml), mouse anti-human CXCR6 (25 µg/ml), mouse anti-human CXCL12 (20 µg/ml) and goat anti-human CXCL16 (20 µg/ml) antibodies. It was demonstrated in Fig. 1 that a specific brown-coloured staining for CXCL16-CXCR6 and CXCL12-CXCR4 in the cytoplasm and membrane of human different pathological types of lung cancer cells. Moderate-to-strong, brown-coloured staining for CXCL16 and CXCR6 was also observed in normal lung tissues, but the positive expression was mainly restricted to the alveolar epithelial cells and inflammatory cells. There was no evidence for nonspecific staining with the control antibody. The pictures were the representative of the experiments. AC: adenocarcinomas; SC: squamous carcinomas; BC: bronchoalveolar carcinoma; Con: normal lung tissues; Villi: human first-trimester villous tissues, as a positive control. Magnification, ×200.

Immunohistochemistry was performed to determine the protein expression of CXCL16 and CXCR6 in human lung caner (33 samples) and normal tissues (5 samples). The results in Fig. 1 clearly demonstrated the coexpression of CXCR6 and CXCL16 protein in human lung cancer tissues. Specific brown-coloured staining for CXCR6 and CXCL16 in the cytoplasm and membrane could be clearly observed in the primary lung cancer cells, but the positive expression rate and staining intensity of CXCR6 was stronger than that of CXCL16. Although moderate-to-strong, brown-coloured staining for CXCL16 and CXCR6 was also observed in normal lung tissues, but the positive expression was mainly restricted to the alveolar epithelial cells and inflammatory cells. There was no evidence for nonspecific staining with the control antibody.

**Figure 2:**
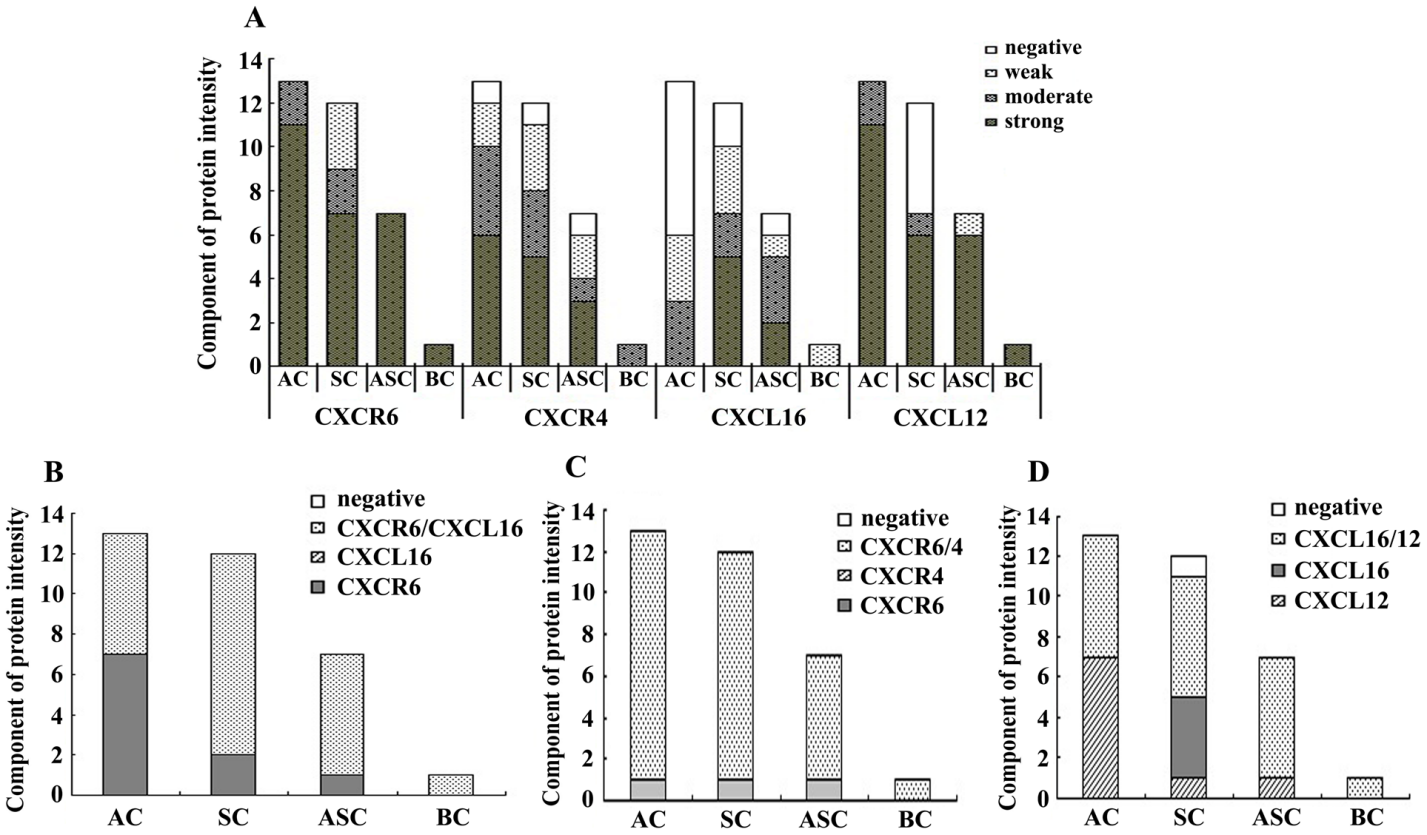
The expression pattern of CXCL16-CXCR6 and CXCL12-CXCR4 in different pathological types of lung cancer. According to the immunostaining intensity score and the percentage of positive cells, the results of immunochemistry analysis were classified as follows: <2, negative expression; 2–3, weak expression; 4–5, moderate expression, and 6–7 as strong expression. AC: adenocarcinomas; SC: squamous carcinomas; ASC: adenosquamous carcinomas; BC: bronchoalveolar carcinoma.

Furthermore, we also analyzed the association of CXCL16-CXCR6 expression pattern with different pathological types of lung cancer. It was demonstrated in Fig. 2 that of the detected 33 lung cancer samples, for CXCR6 protein expression, only 3 SC cases were weak positive and the others were all moderate to strong positive, while for CXCL16, the staining intensity was negative (10), weak (8), moderate (8) and strong (7). Of the 10 CXCL16-negative samples, 7 cases belong to AC, 2 cases belong to SC and 1 case was ASC. Of the 7 CXCL16-strong samples, 5 cases were SC and 2 cases were ASC. The rest of 16 weak to moderate samples were AC (6), SC (5) ASC (4) and BC (1), respectively (Fig. 2).

In the present study we also used CXCL12-CXCR4 as positive control and compared the expression pattern between CXCL16-CXCR6 and CXCL12-CXCR4. As shown in Fig. 1,2 and Table 1 the majority of the samples expressed CXCR4 (30/33) and CXCL12 (28/33) protein, despite of a light difference in the expression intensity. Furthermore, in 33 samples detected, there were 30 cases that co-expressed CXCR6 and CXCR4 protein, and 19 cases that co-expressed CXCL16 and CXCL12 protein.

**Table 1:** Expression intensity of CXCL16/CXCR6 and CXCL12/CXCR4 protein in human lung cancer tissues.

Sample	Type	CXCL16	CXCR6	CXCL12	CXCR4
1	A	−	++++	++++	++
2	A	+	++++	++++	++++
3	A	+	++++	++++	++++
4	A	−	++++	++++	++++
5	A	−	++++	++	++
6	A	−	++++	++++	−
7	A	−	++++	++++	++
8	A	+++	++	++	++
9	A	++	+++	++++	+
10	A	++	++++	++++	++++
11	A	−	++++	++++	++++
12	A	−	++++	++++	+
13	A	+	++++	++++	++++
14	S	++++	+	++++	+++
15	S	++++	++++	−	++++
16	S	+	+	−	−
17	S	++++	++	++++	+
18	S	++++	++++	++++	++++
19	S	+	+	−	+
20	S	++	++++	++++	++
21	S	−	++++	++	+
22	S	+	++++	++++	++++
23	S	++	++++	++++	++++
24	S	++++	++++	−	++++
25	S	−	++	−	++
26	AS	++	++++	++++	++++
27	AS	++++	++++	++++	−
28	AS	++	++++	+	+
29	AS	+	++++	++++	++++
30	AS	++	++++	++++	++++
31	AS	−	++++	++++	+
32	AS	++++	++++	++++	++
33	BAC	+	++++	++++	++
34	Nor	+	+	+	+
35	Nor	++	+	−	+
36	Nor	++	+	−	+
37	Nor	++	+	−	++
38	Nor	++	−	+	−

A: adenocarcinoma, S: squamous cell carcinoma, AS: adeno-squamous carcinoma, BAC: bronchioloalveolar carcinoma, Nor: normal lung tissues, −: negative, +: weak, ++∼+++: moderate, ++++: strong.

### Protein Expression of CXCL16-CXCR6 in Human Lung Cancer Cell Lines

**Figure 3:**
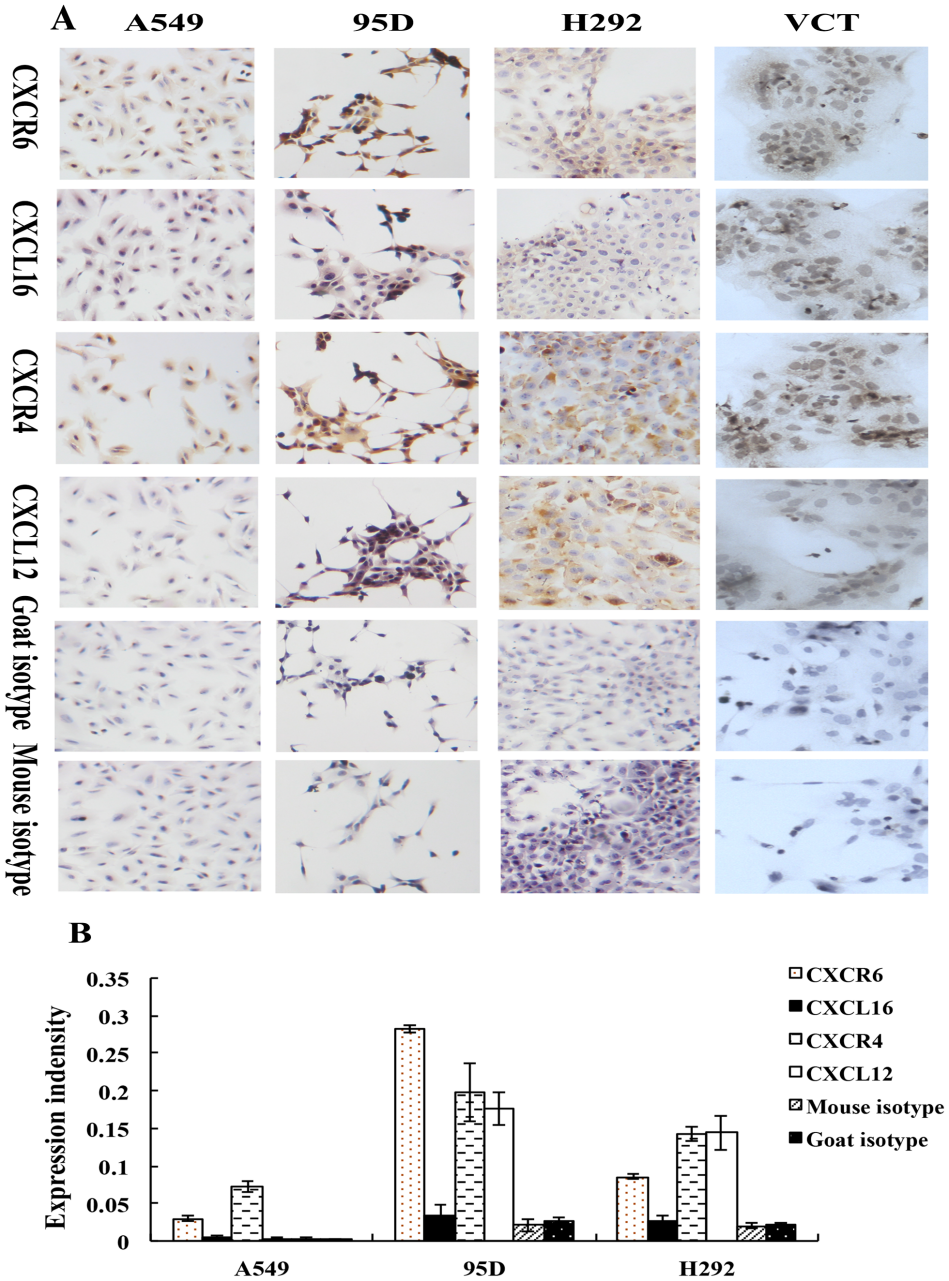
CXCL16-CXCR6 and CXCL12-CXCR4 proteins were co-expressed in human lung cancer cell lines *in vitro*. Immunocytochemistry was conducted to detect the protein expression of CXCL16-CXCR6 and CXCL12-CXCR4 in human lung cancer cell lines. The antibodies used in this experiments were mouse anti-human CXCR4 (25 µg/ml), mouse anti-human CXCR6 (25 µg/ml), mouse anti-human CXCL12 (20 µg/ml) and goat anti-human CXCL16 (20 µg/ml) antibodies. Positive brown-coloured staining for both CXCL16 and CXCR6 was clearly observed in the cytoplasm and cytomembrane of A549, H292 and 95D cells, respectively. Furthermore, CXCL12 and CXCR4 were also co-expressed in the three lung cancer cell lines. No background staining was observed in the isotype control. A: The images were representative of the experiments; B: The relative expression intensity of CXCL16-CXCR6 and CXCL12-CXCR4 in A549, H292 and 95D cells. Tro: human primary cultured trophoblast cells as a positive control. Magnification, ×200.

After confirming the coexpression of CXCL16-CXCR6 protein in human native lung cancer cells, we further analyzed the expression of CXCL16-CXCR6 in human lung cancer cell lines A549, H292 and 95D by immunocytochemistry. As shown in Fig. 3, positive brown-coloured staining for both CXCL16 and CXCR6 could be clearly observed in the cytoplasm and cytomembrane of A549, H292 and 95D cells, respectively. Although there were differences of expression intensity in A549, 95D and H292 cells, the staining for the ligand was all relatively weaker than it’s receptor in three types of cells. Furthermore, the expression pattern of CXCL16-CXCR6 was similar to that of CXCL12-CXCR4, the specific staining for both CXCR4 and CXCL12 could also be observed in three lung cancer cell lines despite of the difference of expression intensity in different cells.

**Figure 4:**
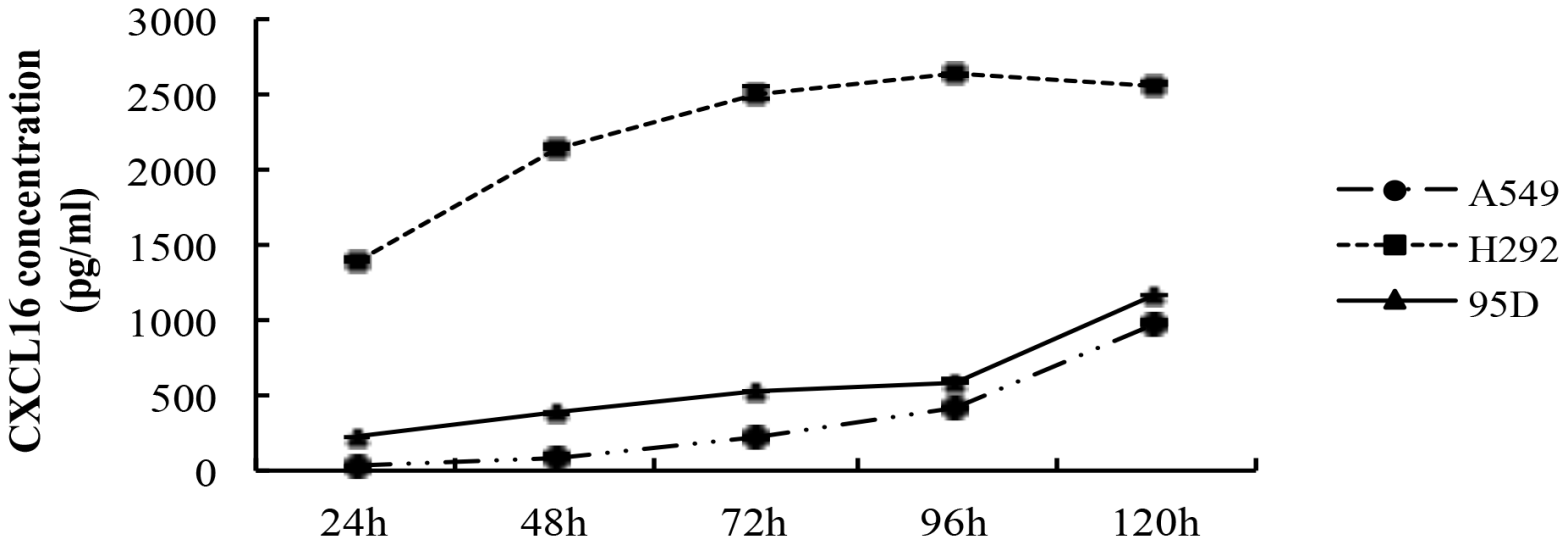
The secretion of CXCL16 by lung cancer cell lines *in vitro*. Digested A549, H292 and 95D cells were seeded in 24-well plates (500 µl/well) at a density of 5×10^5^/ml, respectively. Supernatants of the cell cultures were collected at 24, 36, 48, 60, 72, 96 and 100 h of culture. An ELISA assay was performed to examine the release of the soluble CXCL16 in cultured A549, H292 and 95D cells *in vitro*. As shown in Fig. 4, three kinds of lung cancer cell lines all secreted CXCL16 spontaneously in a time-dependent manner, despites of a difference in the concentration of CXCL16 in the culture medium. The experiments were repeated three times. Error bars depict the standard error of the mean.

Then, an ELISA assay was performed to examine the release of the soluble CXCL16 in cultured A549, H292 and 95D cells *in vitro*. As shown in Fig. 4, three kinds of lung cancer cell lines all secreted CXCL16 spontaneously almost at a constant rate, but there was a slight difference in the concentration of CXCL16 in the culture medium. The amount of CXCL16 produced by H292 cells was the highest among the three cell types. The concentration of CXCL16 in the 24 hours-cultured medium of H292 already reached to 1391.9±13.43 ng/ml and the accumulated concentration of CXCL16 was 2633.2±9.84 and 2566.9±3.1 ng/ml after culture for 96 and 120 h, respectively. Although the production of CXCL16 by A549 cells was relatively less than that of both H292 and 95D cells, the production of CXCL16 was still in a time-dependent manner and the amount of CXCL16 was 977.45±12.85 ng/ml after culture for 120 h. For 95D cells, the concentration of CXCL16 also correlated positively to the culture time and the 120 h production of CXCL16 was 1165.2±12.00 ng/ml.

**Figure 5:**
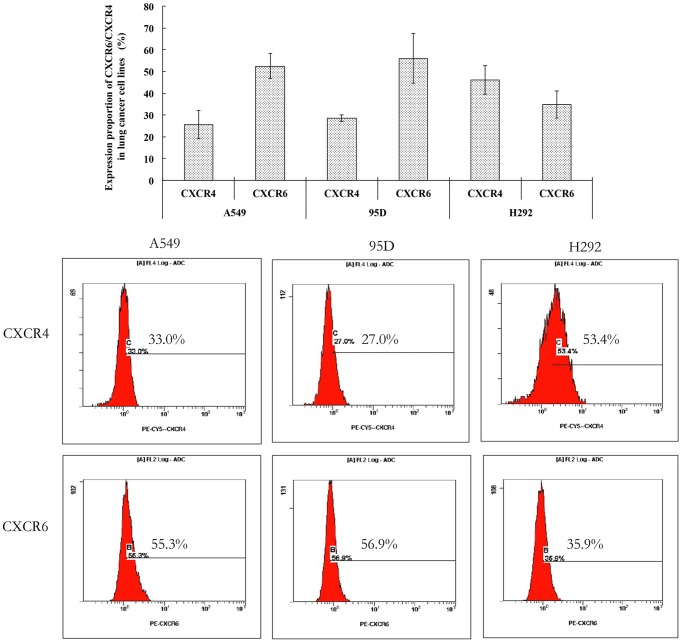
The membrane expression of CXCR6 and CXCR4 in lung cancer cell lines *in vitro*. Flow cytometry (FCM) was used to detect the membrane expression of CXCR6 in lung cancer cell lines. The cells of 1×10^5^ were counted and the dilution ratio of (phycoerythrin)-CXCR6 monoclonal antibody and PE-CY5-CXCR4 monoclonal antibody were 1∶10 and 1∶5, respectively. The histogram demonstrated the average expression proportion of CXCR6 and CXCR4 in A549, H292 and 95D cells, respectively. The FCM pictures were representative of the experiments. The percentage of membrane CXCR6-positive cells in A549, H292 and 95D was 52.4±5.80, 56.03±11.42 and 34.8±6.17, respectively. Furthermore, the membrane expression of CXCR4 was also observed in A549, H292 and 95D cells at the same time. The experiments were repeated three times and the images were representative of the experiments. Error bars depict the standard error of the mean.

Flow cytometry was used to detect the membrane expression of CXCR6 in A549, H292 and 95D cells. Although the proportion of CXCR6 expression by H292 cells was less than that of both A549 (52.4±5.79%) and 95D cells (56.03±11.42%), the average percentage was still 34.87±6.17 (Fig. 5). Furthermore, we also detected the membrane expression of CXCR4 in A549, H292 and 95D cells with flow cytometry. It was found that the percentage of CXCR4-positive cells in A549, H292 and 95D was 25.56±6.44, 46.00±6.52 and 28.57±1.43, respectively. Thus, membrane CXCR6 and CXCR4 were coexpressed in three lung cancer cell lines despite of a slight difference in the positive expression proportion.

### Effects of CXCL16 on PCNA Expression of Lung Cancer Cell Lines *in vitro*

**Figure 6:**
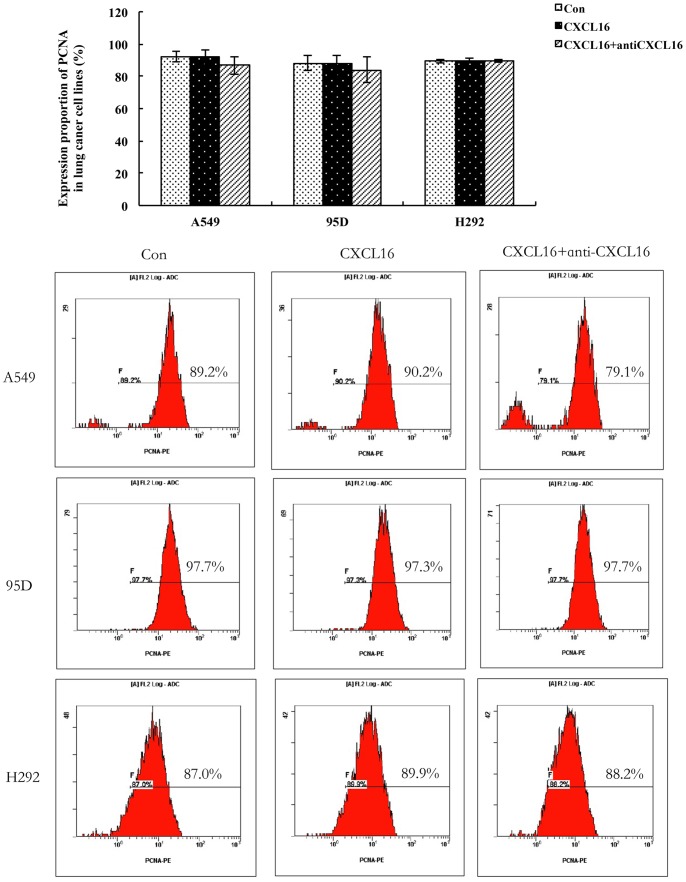
Effects of CXCL16 on PCNA expression of lung cancer cell lines *in vitro*. After starved for 12(100 ng/mL) or a combination of CXCL16 with CXCL16 neutralizing antibody (100 ng/mL) for anther 48 h. Then, the effects of CXCL16 stimulation on PCNA (proliferating cell nuclear antigen) expression was detected by flow cytometry. As shown in Fig. 6, there were no significant changes in PCNA level in various experimental groups (P>0.05, compared with the control). The images are representative of the experiments and the results were reproducible and consistent in three lung cancer cell lines. Error bars depict the standard error of the mean.

After identifying the functional existence of CXCL16 and CXCR6 in A549, 95D and H292, we further observed the effect of CXCL16 stimulation on PCNA expression of these cells. As shown in Fig. 6, there were no significant changes in PCNA level in various experimental groups (P>0.05, compared with the control). The results were highly consistent in three lung cancer cell lines and CXCL16-CXCR6 axis showed no effects on PCNA expression of A549, 95D or H292.

### CXCR6 was Down-regulated by siRNA Techonology

**Figure 7:**
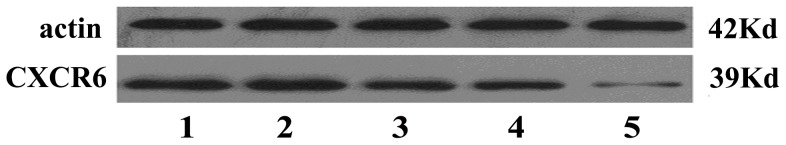
CXCR6 was effectively down-regulated by siRNA techonology. To silence the CXCR6 gene expression, we transfected A549 cells with phU6/GFP/Neo plasmid containing short hairpin RNA (shRNA) molecules targeted against CXCR6, with the usage of Lipofectamine 2000 (Invitrogen). The sequences for three shRNA oligonucleotides were: (CXCR6-2819-1): 5′-ctGAG GAC AAT TCC AAG ACT T-3′ (sense) and 5′-AAG TCT TGG AAT TGT CCT CAG-3′ (Anti-sense); (CXCR6-2820-2) 5′-ctCAC CAT GAT TGT CTG CTA T-3′ (sense) and 5′-ATA GCA GAC AAT CAT GGT GAG-3′ (Anti-sense); (CXCR6-2821-1) 5′-gcTTG CTC ATC TGG GTG ATA T-3′ (sense) and 5′-ATA TCA CCC AGA TGA GCA AGC-3′ (Anti-sense). Efficiency of RNA interference against CXCR6 was validated by western blot. It was shown in Fig. 7 that CXCR6 protein was substantially expressed in A549 cells (bank-control, without any treatment) and the RNA interference technology effectively inhibited CXCR6 expression in A549 cells. Lane 1: Blank-control; Lane 2: RNA-control; Lane 3: CXCR6 shRNA-(2821-1); Lane 4: CXCR6 shRNA-(2820-2); Lane 5: CXCR6 shRNA-(2819-1).

Efficiency of RNA interference against CXCR6 was validated by western blot. It was shown in Fig. 7 that CXCR6 protein was substantially expressed in A549 cells (bank-control, without any treatment) and the RNA interference technology effectively inhibited CXCR6 expression in A549 cells. The silencing efficiency of CXCR6 shRNA-(2819-1) was the best, thus in all the subsequent experiments, we used this siRNA to silence CXCR6 mRNA expression.

### Effects of CXCL16-CXCR6 on A549 Cell Viability *in vitro*

**Figure 8:**
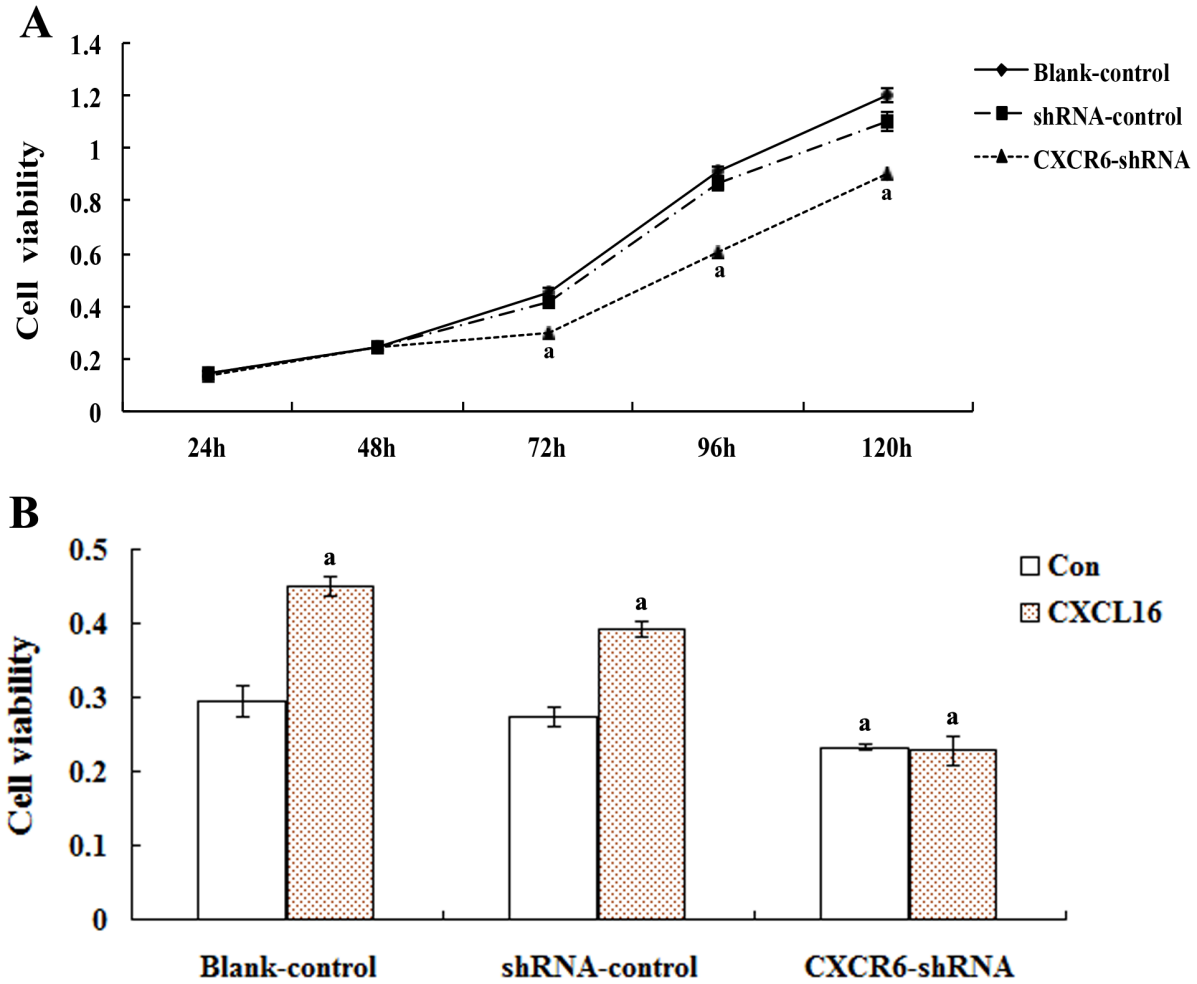
Effects of CXCL16-CXCR6 on A549 cell viability *in vitro*. The MTT assay was applied to evaluate the effects of CXCL16-CXCR6 on cell viability *in vitro*. A: A549 cells from blank-control, shRNA-control and CXCR6-shRNA (2819-1) groups were seeded in 96-well flat-bottom microplates with a density of 2×10^3^ cells/well. The *in vitro* viability was observed at 24, 48, 72, 96 and 120 h respectively. B: After starved with 1640 without FCS for 12 h, the cells from blank-control, shRNA-control or CXCR6-shRNA (2819-1) groups were treated with control medium (1640) or CXCL16 at a concentration of 100 ng/mL for 48 h. Then the *in vitro* viability was recorded by MTT assay. The experiments were repeated three times. Error bars depict the standard error of the mean. ^a^P<0.01, compared with the corresponding control.

MTT assay results in Fig. 8 showed that there was no difference of the *in vitro* viability between the blank-control and shRNA-control. However, compared with the blank-control, the viability of A549 cells from the CXCR6-shRNA (2819-1) groups significantly decreased with the prolongation of culture time (compared with the control, P<0.01 at 72, 96 and 120 h). Moreover, human recombinant CXCL16 was able to increase the *in vitro* viability of A549 cells from the blank-control and shRNA-control (compared with the control, P<0.01), while had no effect on the viability of A549 cells from the CXCR6 down-regulated group (compared with the control, P>0.05).

### CXCL16-CXCR6 Induces Migration and Invasion of Lung Cancer Cell Lines *in vitro*

**Figure 9:**
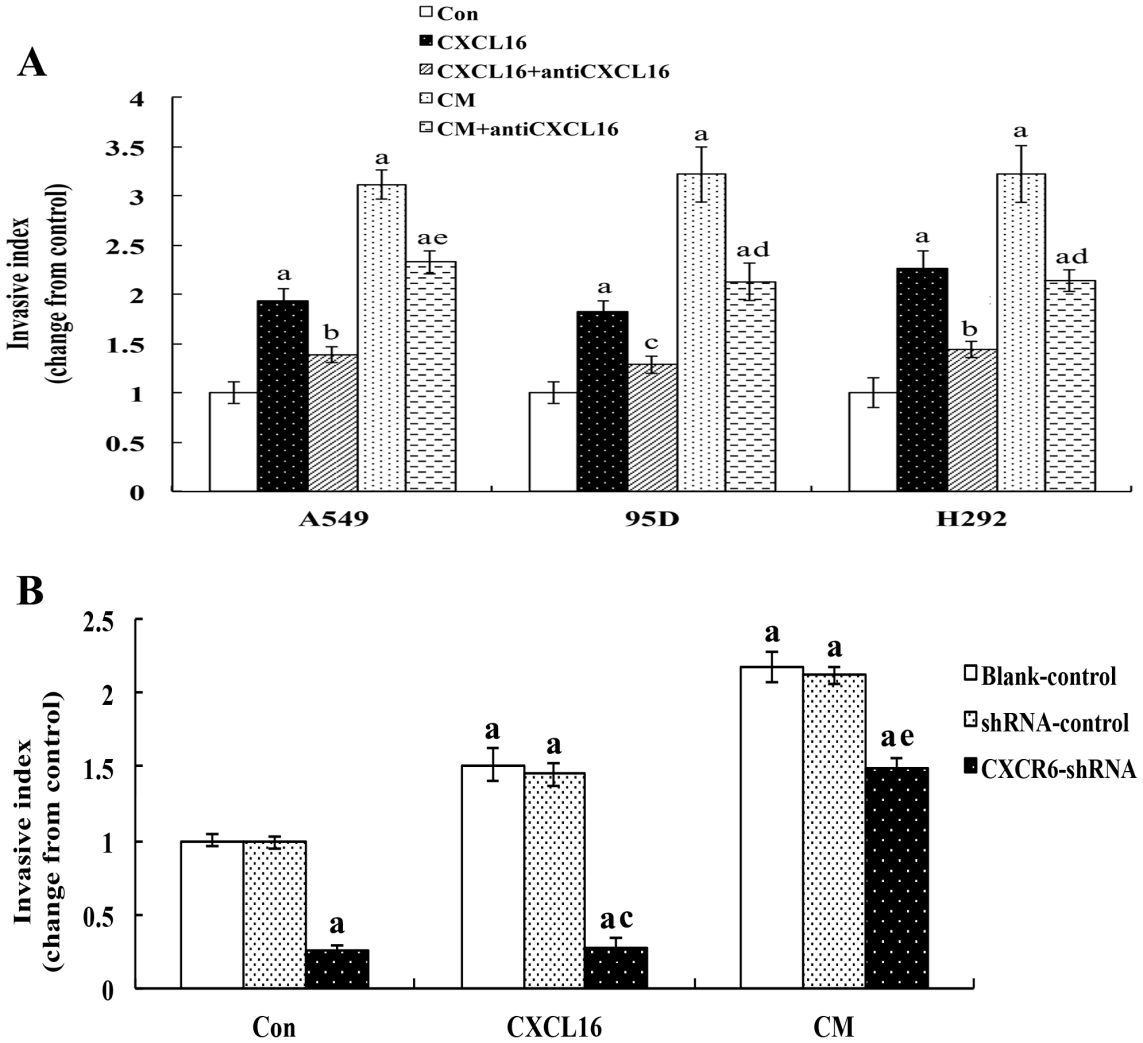
CXCL16 induces migration and invasion of lung cancer cell lines *in vitro*. Invasion assay was employed to investigate the effects of CXCL16-CXCR6 axis on the invasive ability of A549, 95D and H292 cells *in vitro*. Firstly, the isolated A549, H292 or 95D cells (1×10^5^/200 µl serum-free 1640) were plated in the upper chamber, and treated with CM, CXCL16 (100 ng/ml) or a combination of CM or CXCL16 with CXCL16 neutralization antibody(100 ng/mL). Secondly, the A549 cells, from the blank-control, phU6/GFP/Neo-CXCR6 and phU6/GFP/Neo group, were seeded on the upper chamber at a density of (1×10^5^/200 µl serum-free 1640), then treated with CXCL16 (100 ng/ml) or CM. The cells migrated to the lower surface were counted and the invasive index was calculated as the proportion of the migrated cells of the experiment group to that of its own control. Error bars depict the standard error of the mean. Con: the control; CXCL16: treated with 100 ng/ml CXCL16; antiCXCL16: treated with 100 ng/mL CXCL16 neutralizing antibody; CM: conditioned medium for A549, 95D or H292; Blank-control: without any treatment, shRNA-control: phU6/GFP/Neo; CXCR6-shRNA: phU6/GFP/Neo-CXCR6 (2819-1). ^a^P<0.01 compared to the vehicle control; ^b^P<0.05, ^c^P<0.01, compared to the CXCL6 alone; ^d^P<0.05, ^e^P<0.01compared to the CM treatment group.

According to our previous procedure, *in vitro* invasion assay was employed to investigate whether CXCL16-CXCR6 mediated signal has the potential to stimulate the invasion of A549, 95D and H292 cells *in vitro* [19]. Invasion index was calculated to represent the invasive ability of lung cancer cell lines. As shown in Fig. 9, the promoting effect of CXCL16 on invasion of three lung cancer cell lines was highly consistent. CXCL16 at a concentration of 100 ng/mL substantially induced a 1.93-fold, 1.83-fold and 2.26-fold increase in A549, 95D and H292 invasive capacity, respectively and CM from A549, 95D and H292 cells also significantly improved their respective invasive ability. Both neutralizing antibody to CXCL16 and CXCR6 siRNA technology effectively inhibited the increased invasiveness stimulated by CXCL16. However, blocking CXCL16-CXCR6 axis by CXCL16 neutralizing antibody or CXCR6 protein down-regulation only partially blocked the increased invasion of lung cancer cell lines induced by the CM (compared with the control, P<0.01).

## Discussion

The present study using immunohistochemistry confirmed the expression of CXCR6 protein in human primary lung caner cells. The detected 33 samples, including AC, SC, ASC and BC, were all positive for CXCR6 protein, and the expression intensity was mainly moderate to strong. Different from our previous study in human prostate cancer [19], there were a considerable proportion of cases (19/33) that also coexpressed CXCL16, the sole ligand for CXCR6. The unique feature of CXCL16 is its pattern of existence: it can exist both in a transmembrane and soluble form [7], [8], [9]. Membrane-anchored CXCL16 functions as adhension molecule establishing firm arrest of CXCR6 positive cells to CXCL16 expressing cells, which is important for the function of leukocytes and erythrocytes under flow conditions [22]. This kind of non-chemical combination also contributes to the interaction between different types of cells, such as antigen presentation between dendritic cells and T lymphocytes [22]. As a chemoattractant, soluble CXCL16 not only directs migration of CXCR6-positive lymphocytes, but also serves as a powerful stimulator to promote the proliferation and invasion of carcinormal cells via the AKT signaling pathway [18]. Thus, it is reasonable to speculate that lung cancer cells could regulate their own biological functions in a self-regulation manner through the coexpression of CXCL16 and CXCR6. On the one hand, CXCL16-CXCR6 mediated firm adhension is beneficial to tissue localization of carcinormal cells in lung, on the other hand, lung cancer cells could regulate the growth and progression by themselves in an autocrine and paracrine manner, which is similar to the importance of the coexpression of CXCL16 and CXCR6 in human first-trimester villous trophoblast at the materno-fetal interface [13]. Moreover, we also found that different from CXCR6, CXCL16 was mainly expressed in SC and ASC rather than AC. Because of the limitation of the sample size, the relationship of CXCL16-CXCR6 expression pattern to different pathological types and stages needs further investigation.

Preclinical and clinical studies support the viewpoint that chemokine CXCL12 (stromal cell-derived factor, SDF-1) and it’s receptor CXCR4 play a pivotal role in the progression and metastasis of lung cancers, particularly in non-small cell lung cancer (NSCLC) [23], [24], [25]. Thus, in the present study, we also used CXCL12-CXCR4 as positive control and compared the expression pattern of CXCL16-CXCR6 and CXCL12-CXCR4. The results clearly demonstrated that not only CXCR6 and CXCR4 (30/33) but also CXCL12 and CXCL16 (19/33) were coexpressed in both human primary lung caner tissues and lung cancer cell lines. Moreover, in situ tumors, the positive expression proportion and average intensity of CXCR6 were even stronger than that of CXCR4. In fact, besides of CXCL16-CXCR6 and CXCL12-CXCR4, there are some other chemokines and receptors involved in the growth, survival and metastasis of lung cancers [26], [27]. Therefore, it is proposed that just like normal conditions, there might be a complicated chemokines network mediating the biological behavior of tumors. When a signalling pathway is blocked, the malignant cells can activate other alternate signals or molecules, which conforms to the principle of self-preservation via “excessive” biological signals. This helps us to explain, to some extent, why gene silence to a single chemokine or chemokine receptor can not completely block the metastasis of carcinormal cells. Thus, we are still on the way to unveil the intricate role of chemokine/chemokine receptor on tumor initiation and progression.

After confirmed the functional existence of CXCL16 and CXCR6 in A549, H292 and 95D cells by FCM and ELISA, we further performed several *in vitro* experiments to explore the significance of CXCL16-CXCR6 expression in human lung cancer. It was shown that CXCL16 had no effect on the PCNA expression of A549, 95D or H292. However, exogenous CXCL16 and CM effectively promoted the *in vitro* invasiveness of lung cancer cell lines, and the induced invasive ability could be obviously blocked by CXCL16 neutralizing antibody or CXCR6 gene down-regulation. Moreover, it has been shown in our previous MTT assay that CXCL16 remarkably promoted the *in vitro* viability of A549, H292 and 95D [28]. The present study further demonstrated that CXCR6 down-regulation significantly inhibited the increased viability stimulated by CXCL16. It was also confirmed in this research that A549 cells secreted CXCL16 spontaneously and CXCR6 RNAi technology decreased both the *in vitro* viability and invasiveness of A549 in natural conditions. Thus combing our previous study with the present study, we speculated that CXCL16-CXCR6 axis participates in the regulation of in vitro viability and invasion of lung caner cells rather than PCNA expression. However, the neutralizing antibody to CXCL16 or CXCR6 protein down-regulation only partially blocked the increased invasion of A549, 95D and H292 induced by their respective CM, which suggests that besides of CXCL16-CXCR6, there are others signals and molecules regulating the invasion of lung cancer cells.

It is generally accepted that immune cells recruited by chemokines function as anti-tumor precursors in local tissues, however, accumulating evidence shows that inflammation also correlates with tumorigenesis [29], [30]. For example, in prostate cancer, inflammatory cytokines derived from the adjacent infiltrating CXCR6-positive T cells stimulate the production of CXCL16 by cancer cells and CXCL16 enhance the growth of CXCR6-expressing cancers and primary T cells, indicating that CXCL16 and CXCR6 mediate pro-tumorigenic effects between tumorassociated leukocytes and cancer cells in an specific inflammatory milieu [17]. As a unique organ, the lung, especially the bronchial epithelial cell, is an important source of CXCL16 and CXCL16 is beneficial to the recruitment and localization of CXCR6-positive immune cells within the lung, which is an important principle for the protective immunity against quantities of foreign particles inhaled [31], [32]. In the present study, we also observed CXCR6-expressing lymphocytes peripheral to the carcinormal cells in some samples and the expression of CXCL16 and CXCR6 protein in alveolar epithelial cells and inflammatory cells in normal controls. Tumorigenesis is determined not only by the characteristics of neoplastic cells but also by the microenvironment of the specific organ. Therefore, CXCL16-CXCR6 might have bidirectional effects: local immune protection against infection and tumor in normal conditions, and a pro-tumorigenic effect in an inflammation associated immune environment.

In conclusion, we demonstrate that besides of the generally accepted chemokine CXCL12 and it’s receptor CXCR4, CXCL16 and CXCR6 are also co-expressed in human primary lung caner cells and CXCL16-CXCR6 mediated action is involved in the viability and invasiveness of lung caner cell lines *in vitro*. The present results increase our understanding of the significance of CXCL16-CXCR6 axis in the progression and metastasis of lung carcinoma and shed some lights on the targeted cancer therapies.
